# Changes in Gut Microbiota in Peruvian Cattle Genetic Nucleus by Breed and Correlations with Beef Quality

**DOI:** 10.3390/vetsci11120608

**Published:** 2024-11-29

**Authors:** Carlos Quilcate, Richard Estrada, Yolanda Romero, Diorman Rojas, Rolando Mamani, Renán Dilton Hañari-Quispe, Mery Aliaga, Walter Galindo, Héctor V. Vásquez, Jorge L. Maicelo, Carlos I. Arbizu

**Affiliations:** 1 Dirección de Desarrollo Tecnológico Agrario, Instituto Nacional de Innovación Agraria (INIA), Lima 15024, Peru; ceqp2374@yahoo.com (C.Q.); yolanda.bioinfo@gmail.com (Y.R.); diormanr@gmail.com (D.R.); rmamani070@gmail.com (R.M.); 2 Facultad de Medicina Veterinaria y Zootecnia, Universidad Nacional del Altiplano; Puno 21001, Peru; rhanari@unap.edu.pe (R.D.H.-Q.); mlaliaga@unap.edu.pe (M.A.); wgalindo@unap.edu.pe (W.G.); 3 Facultad de Ingeniería Zootecnista, Agronegocios y Biotecnología, Universidad Nacional Toribio Rodríguez de Mendoza de Amazonas (UNTRM), Cl. Higos Urco 342, Chachapoyas 01001, Peru; hvasquez@untrm.edu.pe (H.V.V.); jmaicelo@untrm.edu.pe (J.L.M.); 4 Facultad de Ingeniería y Ciencias Agrarias, Universidad Nacional Toribio Rodríguez de Mendoza de Amazonas (UNTRM), Cl. Higos Urco 342, Chachapoyas 01001, Peru

**Keywords:** gut microbiota, cattle breeds, microbial diversity, meat quality, hematological parameters, host genetics, 16S/18S rRNA

## Abstract

This study evaluated the gut microbiota and meat quality traits in 11 healthy female cattle from the Huaral region of Peru, including 5 Angus, 3 Braunvieh, and 3 F1 Simmental × Braunvieh. All cattle were 18 months old and maintained on a consistent lifelong diet. Meat quality traits, including loin area, fat thickness, muscle depth, and marbling, were assessed in vivo using ultrasonography. Fecal samples were collected for microbiota analysis, and DNA was extracted for 16S and 18S rRNA sequencing to characterize bacterial, fungal, and protist communities. Significant correlations were observed between microbial genera and meat traits: Christensenellaceae R-7 and Alistipes were positively associated with marbling and muscle area, while Rikenellaceae RC9 showed a negative correlation with fat thickness. Among fungi, Candida positively correlated with marbling, while Trichosporon was negatively associated with muscle depth. For protists, Entodinium negatively correlated with fat thickness and marbling. Alpha diversity varied by breed, with Angus showing greater bacterial diversity, and beta diversity analyses indicated a strong breed influence on microbial composition. These findings suggest that microbial composition, shaped by breed and dietary consistency, could serve as an indicator of meat quality, offering insights into gut microbiota’s role in optimizing cattle production.

## Simple Summary

Understanding the gut microbiota—the community of microorganisms residing in the digestive system—of cattle is essential for enhancing animal health and meat quality. This study examined the gut microbiota of different cattle breeds, including Angus, Braunvieh, and Simmental × Braunvieh crossbreeds, to identify how breed-specific microbiota compositions impact health and meat traits. By analyzing the gut bacteria, fungi, and protists through 16S and 18S rRNA sequencing, the research identified significant links between microbial diversity and traits such as fat distribution, muscle quality, and marbling. The findings indicate that both breed and environmental factors, including diet consistency and shape of the gut microbiota, influence cattle productivity and meat quality. This research contributes to more targeted and sustainable cattle farming practices that benefit both producers and consumers.

## 1. Introduction

A healthy gut hosts over 100 trillion microbes, predominantly bacteria (98%), along with fungi, viruses, protists, and archaea. These organisms establish a mutually beneficial relationship with the host, playing essential roles in nutrient uptake, immune response modulation, and maintaining gut barrier integrity [1,2]. The gut microbiota not only synthesizes compounds such as antimicrobial peptides, vitamins, and enzymes but is also shaped by the host species and its health status [3]. Recent research indicates that the host’s genetic makeup can influence the composition of the rumen microbiota, which is linked to rumen metabolites, feed efficiency, and milk production quality [4,5,6,7].

Microbial communities in the intestine are critical for nutrient breakdown, energy extraction, immune modulation, and disease onset [8]. Variations in gut microbiota composition and activity are associated with the overall health and productivity of livestock [9]. Environmental factors and host-related genetic factors significantly shape these microbial communities [10]. Studies have shown considerable variation in the gut microbiome among different animal breeds raised under identical conditions, highlighting breed-specific biomarkers in pigs and broiler chickens [11,12,13]. Furthermore, research into rumen microbial differences among various breeds and crossbreeding in cattle has revealed significant impacts on microbiota and metabolites [14,15,16,17]. These findings underscore the intricate relationship between host genetics, gut microbiota, and phenotypic traits, which are crucial for the livestock industry.

Beef quality is vital for the livestock sector, as it directly influences consumer satisfaction and market value. Genetic and environmental factors play a critical role in determining essential traits such as marbling, tenderness, and flavor, which can optimize breeding practices and enhance production efficiency [18]. Improving beef quality also promotes sustainable management by facilitating efficient resource use and enhancing animal welfare [19]. Key traits like backfat thickness, intramuscular fat content, and tenderness are associated with lipid metabolism and fat accumulation [20,21]. Sensory quality encompasses characteristics such as flavor, color, pH, drip loss, tenderness marbling, and juiciness, all of which are essential for ensuring consumer satisfaction [22]. Additionally, beef quality includes processing attributes, nutritional content, and hygiene standards [23,24,25].

The Angus breed is globally recognized for its high-quality marbled meat, prized for its tenderness and flavor [26,27]. Similarly, the Braunvieh breed serves a dual purpose, providing both milk and high-quality beef with a balanced distribution of fat and tenderness [28,29]. Understanding the unique characteristics of these breeds is essential, as the 16S rRNA gene serves as a key molecular marker for analyzing their ruminal microbiome, offering insights into microbial community composition and diversity [30]. The 18S rRNA marker is also vital for studying the intestinal microbiota of fungi and protists, facilitating accurate detection among eukaryotic organisms [31]. Utilizing advanced sequencing techniques, such as Illumina, allows for thorough examination of microbial communities, including those in low abundance. This approach is crucial for linking microbial diversity with host health, thereby enhancing our understanding of how breed-specific traits influence beef quality and informing the development of targeted probiotic therapies to improve animal health and productivity [32,33].

This study aims to investigate the interaction between gut microbiota and meat quality traits in different cattle breeds within a genetic core. Angus, Braunvieh, and Simmental-Brahman crosses will be analyzed, characterizing microbial diversity by 16S and 18S ribosomal gene sequencing. Correlations between breed-specific microbiota and meat quality traits such as marbling, tenderness, and fat composition will be assessed. Furthermore, the influence of genetic and environmental factors on these microbial communities will be explored. Our hypothesis suggests that microbial richness and composition will vary significantly by breed, with relevant correlations between gut microbiota diversity and meat quality parameters.

## 2. Materials and Methods

### 2.1. Animal Experiment and Sample Collection

In the Huaral region of Lima, situated at an elevation of 128 m above sea level (coordinates 11°31′18″ S and 77°14′06″ W), a total of 11 fecal samples were collected from healthy cattle all females of 3 different breeds (5 Angus and 3 Braunvieh) and 3 crossbreeds F1(SMxBR) from the EEA Donoso. All cattle were 18 months old with an average body weight of 497 kg and had been maintained on a consistent diet throughout their entire lives with no adaptation period required as their diet was never altered. Feeding was conducted three times daily, with portions proportionally distributed across the total diet: 8:00 am, 12:00 pm, and 5:00 pm. Water was provided ad libitum through troughs controlled by float valves. The pens featured smooth metal structures composed of round tubing.

The diet at the Donoso EEA primarily consists of a specific type of forage, supplemented with additives (Table 1) and balanced feed. The composition of the balanced feed was sourced from Montana https://lc.cx/rV2bC8 (accessed on 25 November 2024) and Molinorte https://lc.cx/2eFqtq (accessed on 25 November 2024) and is detailed in Table 2 and Supplementary Table S1. Fecal samples (50 g each) were aseptically obtained from the rectum of each animal using sterile disposable obstetric gloves in the morning on a single day of the experiment, after their first feeding at eleven o’clock. These samples were promptly immediately transported to the laboratory under liquid nitrogen and stored at −80 °C prior to DNA extraction. The veterinary team at the EEA Donoso Genetic Center in Huaral regularly monitored the animals, conducting parasitological examinations that confirmed the absence of cysts, oocysts, or larvae. Routine veterinary evaluations, including physical exams, medical history assessments, and laboratory tests, were performed to ensure high health standards. As a result, there were no sick animals in the genetic nucleus. This study was carried out in accordance with Peruvian National Law No. 30407: “Animal Protection and Welfare”.

### 2.2. Meat Quality Traits Detection

To acquire the ultrasound images, the cattle were immobilized and secured by the head in a squeeze chute. The imaging sites were identified through physical palpation to ensure precise determination of the scanning locations. The animals were manually restrained, ensuring that no abnormal conditions arose that could cause stress. Ultrasound scanning was performed only when the animals were in a relaxed posture, allowing for accurate measurements. Ultrasound imaging was performed to assess the loin skin thickness (GPL), loin fat thickness (GGL), loin thickness (GL), hip skin thickness (GPC), hip fat thickness (GGC), marbled beef ultrasonography right buttock (NMG1), marbled beef ultrasonography right buttock (NMG2), and loin area (AL), were measure in vivo using an ESAOTE (Esaote Pie Medical, Aquila Vet model, with a 6 MHz linear transducer, Maastrich, The Netherlands) ultrasound machine equipped with an APS 3.5 MHz transducer. To enhance image clarity, the measurement site was shaved, cleaned, and lubricated with vegetable oil, and a soft material guide was employed to improve contact between the transducer and the animal’s curved body surfaces. The weight of the animal was measured at the same time as the ultrasonography. This procedure was carried out in the afternoon on the same day as the fecal sample collection.

### 2.3. Analyses of Blood Parameters

Blood was drawn from the jugular vein of each animal, and 11 samples in total were evaluated for parameters such as red blood cell count, white blood cell count, and platelet levels using the Dx^®^ hematology analyzer (IDEXX Laboratories, Westbrook, MA, USA). Additionally, 11 plasma samples were analyzed for triglyceride levels using the Beckman-CX4 automatic biochemical analyzer (Beckman Coulter, Inc., Brea, CA, USA). Detailed information on the variables analyzed is provided in Supplementary Table S2.

### 2.4. DNA Extraction and Sequencing

The genomic DNA from each fecal sample was extracted utilizing the QIAamp DNA Stool Mini Kit (Qiagen, Valencia, CA, USA). To construct the Illumina amplicon sequencing library, approximately 10 ng of DNA from each sample were used for PCR amplification with primers 515F/806R for 16S rRNA and 528F/706R for 18S rRNA were performed according to a previously described protocol for PCR conditions to 16S rRNA [34] (Treven et al., 2019) and 18S rRNA [35] (kARST et al., 2018). Sequencing libraries were prepared using the Illumina TruSeq DNA PCR-Free Library Preparation Kit (Illumina, San Diego, CA, USA) as per the protocol, which included the addition of index sequences. The quality of the libraries was assessed with a Qubit 2.0 fluorometer (Thermo Scientific, Waltham, MA, USA). The validated libraries were then sequenced by the sequencing service of Novogene (San Diego, CA, USA) on the Illumina NovaSeq 6000 platform with 250 bp paired-end reads (Illumina Inc., San Diego, CA, USA) following the manufacturer’s instructions.

### 2.5. Bioinformatics Analysis

The paired-end FASTQ sequences underwent trimming and processing prior to identifying Amplicon Sequence Variants (ASVs). The classification of ASVs and subsequent diversity analyses were carried out using the QIIME2 [36] platform following established protocols. From the initial trimming stage to the microbiome diversity analysis, each step adhered to previously validated methods. ASVs were matched against the SILVA v138.1 reference database, with clustering performed at a 97% similarity threshold. Taxonomic assignment up to the genus level was performed using the SILVA v138.1 database, with taxonomy naming based on the SILVA phylogenetic classification system https://www.arb-silva.de/browser/ssu/ (accessed on 25 November 2024).

### 2.6. Statistics Analysis

The dataset was processed and analyzed in R software [37] (version 4.1.1) using the Phyloseq [38], Microeco [39], and MicrobiotaProcess [40] packages. Rarefaction curves were generated to assess sequencing depth across all samples. For alpha diversity, indices such as Observed, Chao1, ACE, Pielou, Simpson, and Shannon were calculated to evaluate microbial diversity within bacterial, fungal, and protist communities. Beta diversity, representing the differences between microbial communities across samples, was analyzed using Bray–Curtis and Jaccard distances and visualized via Principal Coordinate Analysis (PCoA). A one-way PERMANOVA test with 9999 permutations was conducted to assess group-level differences in community composition. Correlation analyses using Spearman’s rank method, with False Discovery Rate (FDR) correction, were performed to explore the relationships between meat quality parameters and microbial genera. Additionally, Linear Discriminant Analysis (LDA) with the LEfSe method was employed to identify microbial biomarkers of significance, focusing on both statistical and biological relevance. Taxa were displayed if LDA values > 2.0 and the *p* value was below 0.05. Correlation analyses using Spearman’s rank method were performed to explore the relationships between blood and meat quality parameters and alpha diversity indices. Mantel tests, with 999 permutations, were employed to examine the associations between environmental variables and microbial community structure.

## 3. Results

The meat quality parameters for the three cattle breeds—Angus, Braunvieh, and F1(SMxBR)—exhibit significant variation. The Angus breed exhibited average (Table 3) values of 6.31 for GPL, 4.12 for GGL, 6.35 for GL, 55.14 for AL, 4.00 for GPC, 2.83 for GGC, 162.20 for NGM1, and 131.16 for NGM2. In contrast, the Braunvieh breed demonstrated higher average values for most parameters, with 6.72 for GPL, 3.32 for GGL, 6.58 for GL, 61.13 for AL, 4.51 for GPC, 2.23 for GGC, 107.19 for NGM1, and 91.71 for NGM2. In comparison, the F1(SMxBR) breed displayed lower average values, recording 6.14 for GPL, 3.18 for GGL, 5.34 for GL, 46.35 for AL, 3.56 for GPC, 1.24 for GGC, 83.52 for NGM1, and 71.16 for NGM2.

### 3.1. Analysis of Effect of Breed on the Gut Microbiota Diversity and Composition

**Figure 1:**
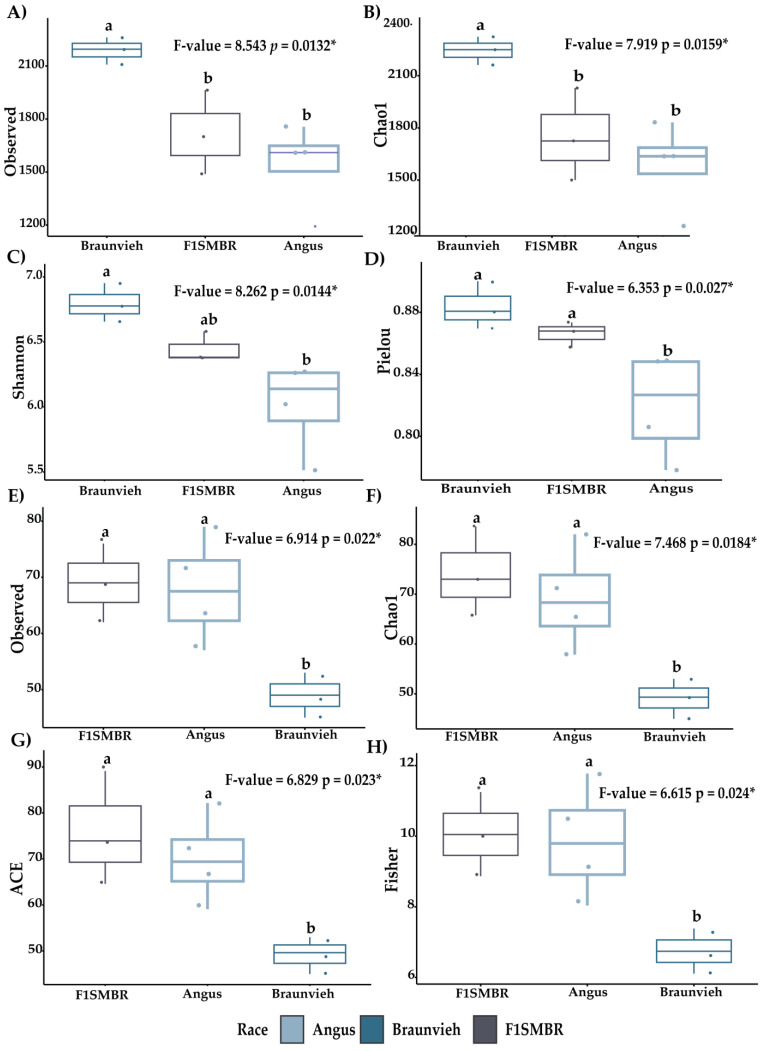
Alpha diversity metrics of bacteria and fungi were evaluated in cattle in three breeds. Diversity indices exhibited include ACE, Chao1, Observe, Pielou, Shannon, and Fisher. (**A**–**D**) Alpha diversity of bacteria (**E**–**H**) Alpha diversity of fungi. Different letters indicate statistically significant tests. * *p* < 0.05. p = *p-*value.

The ANOVA test was conducted to assess the significance of alpha diversity comparisons (Figure 1). Regarding bacterial alpha diversity, Braunvieh exhibited higher diversity than F1SMBR and Angus across multiple indices: Observed (*p* = 0.0132), Chao1 (*p* = 0.0159), Shannon (*p* = 0.0144), and Pielou (*p* = 0.0027). For fungal alpha diversity, F1SMBR demonstrated greater diversity compared to Angus and Braunvieh in Observed (*p* = 0.022), Chao1 (*p* = 0.0184), ACE (*p* = 0.023), and Fisher (*p* = 0.024). In the case of protistan alpha diversity (Figure S2), F1SMBR displayed higher values than both Angus and Braunvieh in Shannon (*p* = 0.025), Simpson (*p* = 0.021), and Pielou (*p* = 0.017).

**Figure 2:**
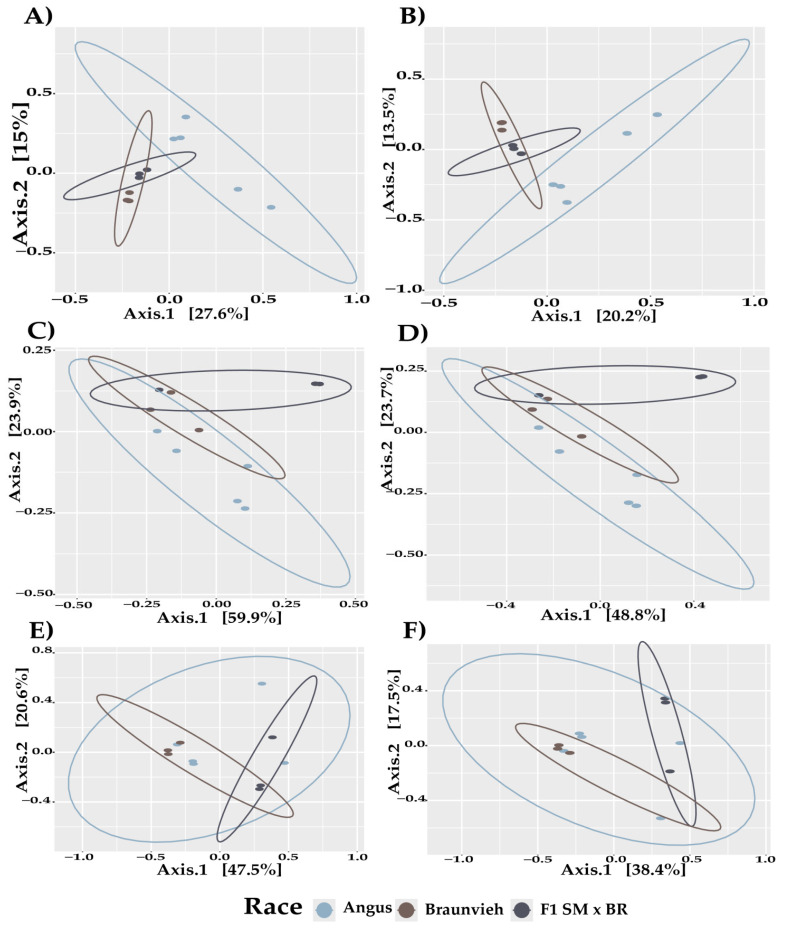
Bray–Curtis and Jaccard analysis of beta diversity in the different breeds of cattle. (**A**) Bray–Curtis of bacteria (**B**) Jaccard of bacteria (**C**) Bray–Curtis of fungi. (**D**) Fungi Jaccard (**E**) Protist Bray–Curtis (**F**) Protist Jaccard.

The analysis evaluates the effect of cattle breed on microbial communities (bacteria, fungi, and protists) using Bray–Curtis and Jaccard dissimilarity indices (Figure 2). For bacteria, the breed factor had a highly significant influence, with a *p*-value of 0.001 for both Bray–Curtis and Jaccard indices, indicating a strong association between breed and bacterial community composition (Table 4). The residuals for the Jaccard index were also significant (*p* = 0.0003).

For fungi, the breed effect was statistically significant, with *p*-values of 0.0053 for Bray–Curtis and 0.0044 for Jaccard, suggesting that fungal community structure is influenced by the cattle breed (Table 4).

For protists, significant effects were observed, with *p*-values of 0.019 for Bray–Curtis and 0.0156 for Jaccard (Table 4), indicating that the protist community composition is related to the breed of cattle. These significance levels suggest that microbial community compositions across bacteria, fungi, and protists vary substantially depending on cattle breed.

Venn diagrams illustrate ASVs of bacteria, fungi and protists, both unique and shared, among cattle of different breeds (Figure S3). For bacterial ASVs (Figure S3A), 1354 are common to all breeds, while 1455 are unique to Angus, 834 to F1(SMxBR) and 1680 to Braunvieh. In the fungal ASVs (Figure S3B), 43 are common to all breeds, with 82 unique to Angus, 45 to F1(SMxBR), and 23 to Braunvieh. For protist ASVs (Figure S3C), 38 are common to all breeds, while 92 are unique to Angus, 72 to F1(SMxBR), and 23 to Braunvieh.

### 3.2. Effect of Breed on the Gut Microbiota Taxonomy

**Figure 3:**
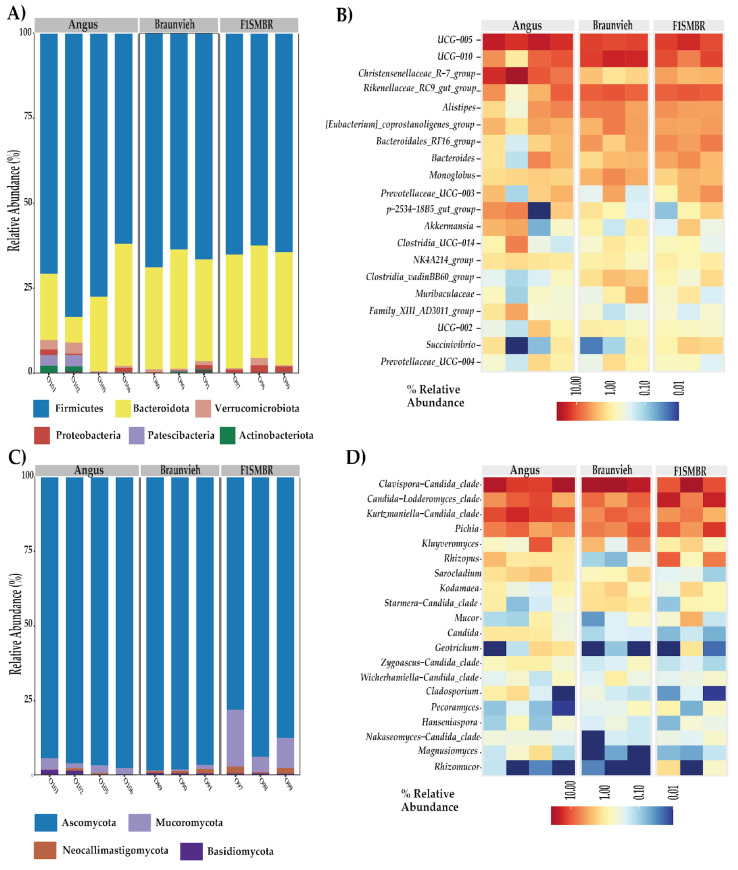
Relative abundances in the gut microbiota at the phylum and genus level in different cattle breeds. (**A**) Bar graph analysis illustrates the abundance of bacterial phyla in each breed. (**B**) Heat map with the main bacterial abundances of the 20 genera in each breed. (**C**) Bar graph analysis illustrates the abundance of fungal phyla in each breed. (**D**) Heat map with the main abundances of fungi of 20 genera in each breed.

At the phylum level, Firmicutes were the dominant bacterial phylum (Figure 3A), comprising 69% in Angus, 67% in Braunvieh and 65% in F1(SMxBR). Bacteroidota accounted for 26%, 31%, and 31% in Angus, Braunvieh and F1(SMxBR), respectively. All other bacterial taxa collectively represented 5%. The dominant fungal phylum (Figure 3C) was Ascomycota, with representations of 95% in Angus, 93% in Braunvieh and 88% in F1(SMxBR). All other fungal phyla collectively represented 5%. Among protists (Figure S4A), the dominant phyla were Ciliophora and Incertae Sedis. Ciliophora constituted 46% in Angus, 85% in Braunvieh and 22% in F1(SMxBR). Incertae Sedis were represented by 36% in Angus, 12% in Braunvieh and 55% in F1(SMxBR). Chlorophyta represented 7%, 2% and 12% in Angus, Braunvieh and F1(SMxBR), respectively. Apicomplexa accounted for 9%, 1% and 7% in Angus, Braunvieh and F1(SMxBR), respectively. All other protist phyla collectively represented 5%.

The heatmap depicts the relative abundance of genera among the analyzed breeds (Figure 3B,D). The 20 most abundant genera were observed. In the bacterial characterization composition at the genus level (Figure 3B), the most abundant genera observed were *UCG-005*, *UCG-010*, *Rikenellaceae_RC9_gut_group*, *Christensenellaceae_R-7_group*, and *Alistipes*. Regarding fungal composition (Figure 3D), the predominant genera were *Clavispora-Candida_clade, Candida-Lodderomyces_clade, Kurtzmaniella-Candida_clade*, and *Pichia*. In the protist composition (Figure S4B), the most abundant genera were *Trichostomatia, Blastocystis*, *Trebouxiophyceae*, and *Gregarina*.

### 3.3. Relationship Between Gut Microbiota and Beef Quality Variables

**Figure 4:**
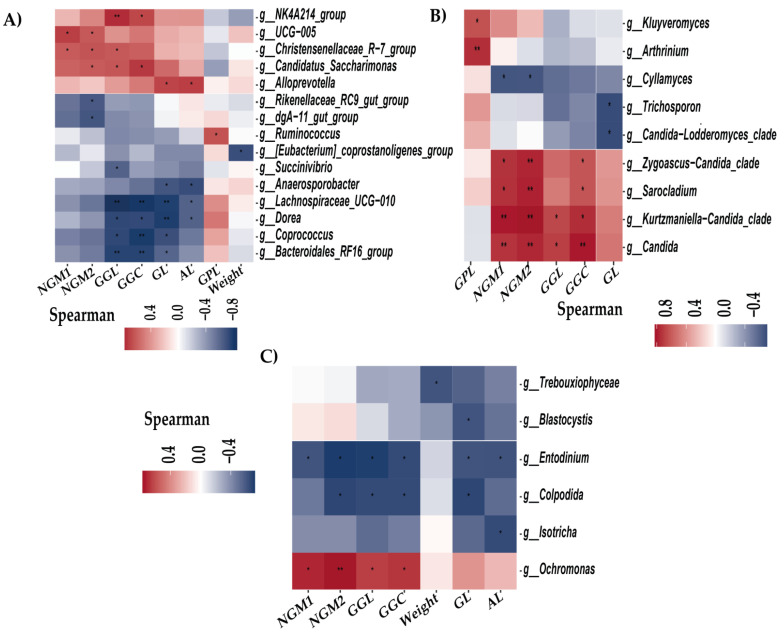
Heat map of Spearman correlation analysis between the genera of intestinal microbes and meat variables. * *p* < 0.05; ** *p* < 0.01 (**A**) Heat map of bacterial genera. (**B**) Heat map of fungi genera. (**C**) Heat map of protist genera.

The Spearman correlation analysis between meat quality variables and the relative abundances of intestinal microbiota genera identified several significant associations (Figure 4). Among bacterial genera (Figure 4A), NGM1 and NGM2 were positively correlated with UCG-005 and Christensenellaceae_R-7_group, while NGM2 exhibited negative correlations with Rikenelaceae_RC9_gut_group and dgA-11_gut_group, alongside a positive correlation with Candidatus_Saccharimonas. GGL and GGC were negatively correlated with Lachnospiraceae_UCG-010, Dorea, Coprococcus, and Bacteroidales_RF16_group, while GGL presented positive correlations with NK4A214_group and Christensenellaceae_R-7_group. GLL exhibited a significant negative correlation with Succinivibrio and a positive one with Christensenellaceae_R-7_group. GL was negatively correlated with Lachnospiraceae_UCG-010, Dorea, Coprococcus, Bacteroidales_RF16_group, and Anaerosporobacter, while both GL and AL were positively correlated with Alloprevotella. AL also demonstrated negative correlations with Anaerosporobacter, Lachnospiraceae_UCG-010, and Dorea. GPL was positively correlated with Ruminococcus, while body weight exhibited a negative correlation with Eubacterium_coprostanoligenes_group and a positive correlation with Akkermansia.

Regarding fungal genera (Figure 4B), GPL was positively correlated with Kluyveromyces and Arthrinium. NGM1, NGM2, and GGC demonstrated positive correlations with Candida, Kurtzmaniella-Candida_clade, Sarocladium, and Zygoascus-Candida_clade, while NGM1 and NGM2 had negative correlations with Cyllamyces. GGL was positively correlated with Candida and Kurtzmaniella-Candida_clade, whereas GL was negatively correlated with Trichosporon and Candida-Lodderomyces_clade.

For protist genera (Figure 4C), NGM1, NGM2, GGL, and GGC exhibited positive correlations with Ochromonas and negative correlations with Entodinium. NGM2, GGL, and GGC also showed negative correlations with Colpodida. Body weight was negatively correlated with Trebouxiophycecae, while GL demonstrated negative correlations with Colpodida, Entodinium, and Blastocystis. Lastly, AL exhibited negative correlations with Isotricha and Entodinium.

### 3.4. Biomarkers Identification for Different Breed

**Figure 5:**
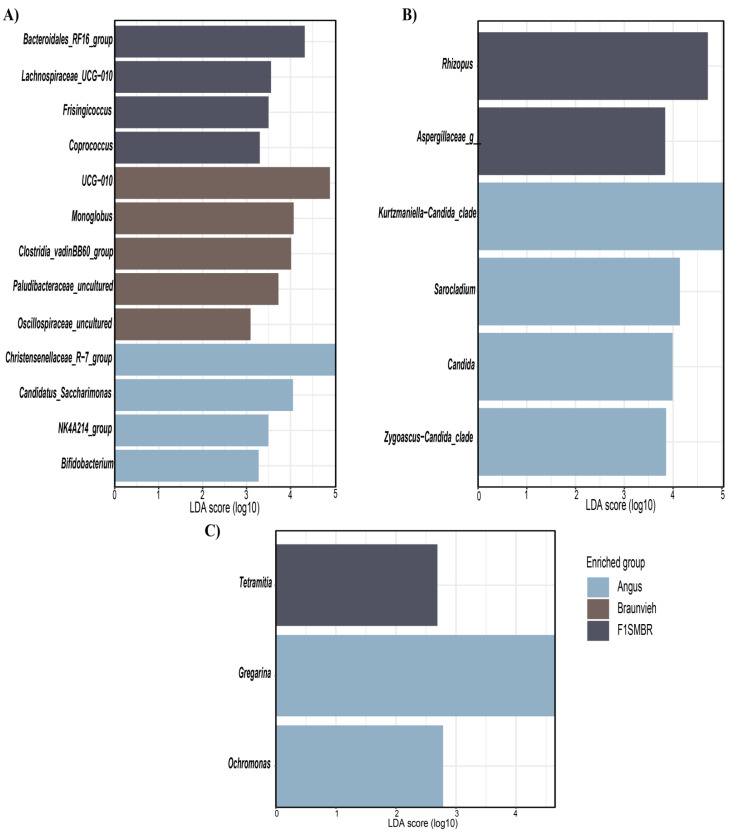
LEfSe analysis of the intestinal microbiome in different breeds. (**A**) LEfSe of bacteria. (**B**) LEfSe of fungi. (**C**) LEfSe of protist.

To identify the bacterial genera associated with each breed, a comparative evaluation of gut microbiota composition was performed. The Linear Discriminant Analysis Effect Size (LEfSe) method was employed to identify the most distinct genera. The most notable differences in taxa were highlighted based on their LDA scores, focusing exclusively on the genus level (Figure 5).

In Angus cattle, the bacterial genera identified as biomarkers (Figure 5A) included *Christensenellaceae_R-7_group, Candidatus_Saccharimonas, NK4A214_group*, and *Bifidobacterium*. In Braunvieh, the identified biomarkers were *UCG-010, Monoglobus, Clostridia_vadinBB60_group, Paludibacteraceae_uncultured*, and *Oscillospiraceae_uncultured*. In F1(SMxBR), the bacterial biomarkers included *Bacteroidales_RF16_group, Lachnospiraceae_UCG-010, Frisingicoccus*, and *Coprococcus*. For fungal biomarkers (Figure 5B), *Zygoascus-Candida_clade, Candida, Sarocladium*, and *Kurtzmaniella-Candida_clade* were identified in Angus, while *Aspergillaceae* sp. and *Rhizopus* were detected in F1(SMxBR). Regarding protist biomarkers (Figure 5C), *Gregarina* and *Ochromonas* were identified in Angus, and *Tetramitia* was observed in F1(SMxBR).

### 3.5. Breed Relationship of Alpha/Beta Diversity with Variables

The variables, hematological and quality meat, were analyzed using the Kruskal–Wallis test, with breed as the variable of interest (Table S3). Only significant parameters for breeds included GL, GGC, NGM1, and NGM2 (Table S4). Subsequently, Bonferroni post hoc analysis was conducted. For GL, the comparison between Angus and F1(SMxBR) yielded a significance of 0.049. In the case of GGC, the comparison between Angus and F1(SMxBR) had a significance of 0.015. For NGM1, the comparison between Angus and F1(SMxBR) was significant at 0.0397. Finally, for NGM2, the comparison between Angus and F1(SMxBR) was significant at 0.0268 (Table S5).

**Figure 6:**
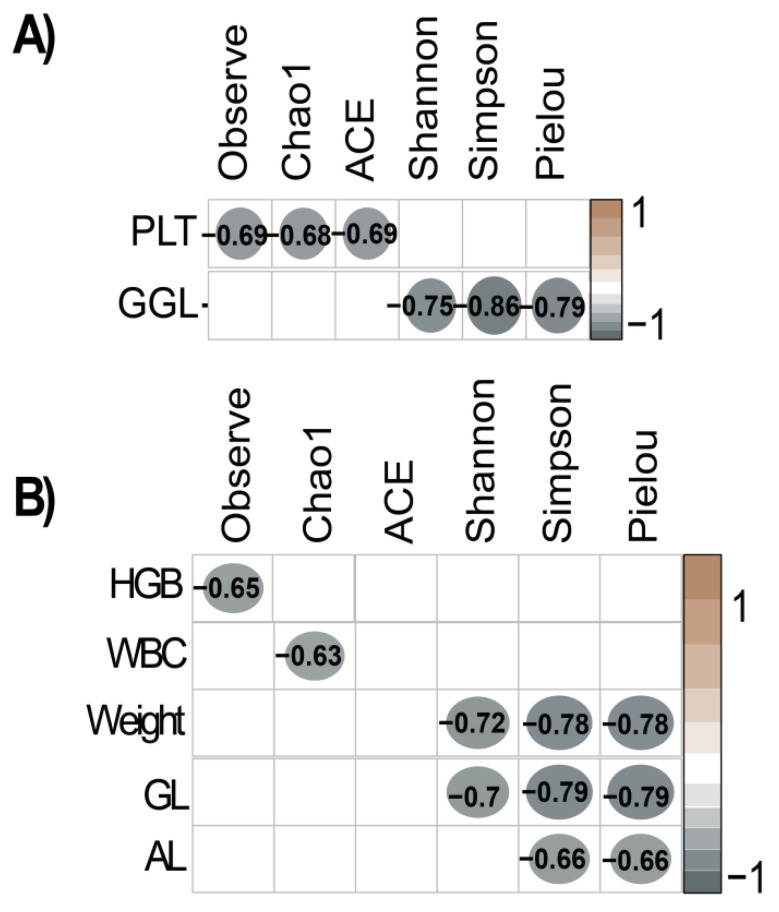
Correlations between the alpha diversity of the intestinal microbiota and hematological and meat variables. (**A**) Spearman correlation of alpha diversity of bacteria. (**B**) Spearman correlation of protist alpha diversity.

The relationship between variables and alpha diversity indices for bacteria and protists was assessed through Spearman correlation analysis (Figure 6). For bacteria (Figure 6A), the Observed, Chao1, and ACE indices exhibited significant negative correlations with PLT, while GGL demonstrated significant negative correlations with the Shannon, Simpson, and Pielou indices.

For protists (Figure 6B), the Observed index was significantly negatively correlated with HGB, and the Chao1 index showed a significant negative correlation with WBC. Additionally, the Shannon, Simpson, and Pielou indices were significantly negatively correlated with both weight and GL. AL exhibited significant negative correlations with the Simpson and Pielou indices.

The Mantel and Partial Mantel tests were conducted to evaluate the correlations between variables and microbial diversity using Bray and Jaccard indices for bacteria, fungi, and protists (Table 5). For bacteria, significant correlations with the Bray index were observed for GGL (*p* = 0.019), NMG1 (*p* = 0.007), and NGM2 (*p* = 0.019). The Partial Mantel test also indicated significant associations for GGL (*p* = 0.017) and NMG1 (*p* = 0.02). Regarding the Jaccard index, significant correlations were detected for GGL (*p* = 0.028), NMG1 (*p* = 0.026), and NGM2 (*p* = 0.016). The Partial Mantel test confirmed the significance for GGL (*p* = 0.037) and NMG1 (*p* = 0.003).

For fungi, the Bray index exhibited significant correlations with RBC (*p* = 0.01), MCV (*p* = 0.012), and MCHC (*p* = 0.008). The Partial Mantel test supported the correlations for RBC (*p* = 0.009), MCV (*p* = 0.005), and MCHC (*p* = 0.004). In the case of the Jaccard index, significant associations were observed for MCHC (*p* = 0.008), NGM1 (*p* = 0.024), NGM2 (*p* = 0.03), and GGC (*p* = 0.011), with further validation by the Partial Mantel test for GGC (*p* = 0.007).

For protists, the Bray index demonstrated significant correlations with NEU (*p* = 0.044) and SEG (*p* = 0.043), and the Partial Mantel test indicated significance for NEU (*p* = 0.031) and SEG (*p* = 0.045). For the Jaccard index, significant correlations were identified for NGM1 (*p* = 0.001), NGM2 (*p* = 0.001), and PLT (*p* = 0.025), with the Partial Mantel test confirming the significance for NGM1 (*p* = 0.004), NGM2 (*p* = 0.002), and PLT (*p* = 0.036).

## 4. Discussion

In this study, it is evident that the intestinal microbiota of cattle is influenced by genetic factors, as documented in previous studies with pigs [41,42]. These studies revealed significant correlations between the microbial composition in the pig intestine and meat quality parameters, emphasizing the importance of understanding interactions between the microbiota and health or production indicators [41]. Consistent with these findings, our results demonstrate that host genetics significantly influence the diversity and composition of the gut microbiota, as evidenced by notable variations in alpha diversity indices across cattle breeds. Notably, Angus cattle exhibited greater microbial diversity. These parallels between cattle and pigs suggest that cattle breeds may modulate the gut microbiota in a similar manner, with implications for genetic improvement in livestock [42]. For instance, research indicates that host genetics influence the rumen microbiota, with factors such as breed, sex, and diet contributing to microbial variation in beef cattle. Additionally, differences in meat quality between Angus and Chinese Simmental cattle have been linked to variations in gut microbiota composition, highlighting the role of genetic factors in shaping microbial communities and their impact on meat quality. These findings reinforce the importance of considering host genetics when developing strategies to manipulate the gut microbiota, as such approaches have the potential to enhance both animal health and production outcomes.

Clinical studies were carried out that indicated normal hematological values in cattle, indicating that the animals were healthy [43]. Additionally, meat quality analyses demonstrated that parameters such as marbling and fat content were within acceptable ranges [44].

The race exhibited a significant correlation with alpha diversity of the intestinal microbiota, consistent with previous findings in bacteria [3,45], fungi [2,46], and protists [47,48,49]. These studies highlight the pivotal influence of genetic and environmental factors in shaping the microbial communities within the gut. The observed differences in alpha diversity among various races suggest that host genetics significantly influence the richness and evenness of gut microbiota. This aligns with evidence from other animal studies, where specific breeds demonstrate distinct microbial profiles due to inherent genetic traits and adaptive responses to their environments [2,3]. In pigs, alpha diversity of gut eukaryotic communities, including fungi and protists, exhibited low but notable heritability, indicating a limited genetic influence on the diversity of these communities [49]. Similarly, in cattle, different breeds exhibit significant variations in rumen bacterial diversity, which can affect nutrient digestion and energy harvest [50,51].

This study identified significant variations in gut microbiota among breeds through beta diversity analysis. Alterations in livestock microbiota associated with breed differences are extensively documented, with specific breeds demonstrating distinct microbial communities [3,51]. For example, studies have indicated that bacterial diversity in the rumen varies among breeds, with some breeds exhibiting higher diversity associated with lower nutrient utilization efficiency [7]. Similarly, the influence of breed was observed in protist and fungal communities, similar to findings in bulls [48], pigs [49], and humans [47]. These studies underscore the crucial role of breed in shaping gut microbiota, as they highlight significant variations in microbiota composition linked to these biological factors, suggesting their profound impact on the configuration of microbial communities across different species. Understanding these correlations is essential for developing targeted strategies to manipulate gut microbiota for improving health and disease management in different breeds.

All the analyzed breeds exhibited the same predominant bacterial phyla, Firmicutes and Bacteroidota, which is consistent with previous studies in cattle and other ruminants, such as goats [4,52], alpacas [53,54], and cattle [3,55]. Firmicutes, known for their role in fermentation of complex carbohydrates and the production of short-chain fatty acids, are crucial for the digestive efficiency of ruminants [56]. Bacteroidota, on the other hand, are essential for breaking down plant polysaccharides and producing volatile fatty acids [57]. Similarly, all breeds exhibited Ascomycota as the predominant fungal phylum, followed by Mucoromycota, corroborating previous studies in cattle [58,59], alpacas [53], and goats [46]. Ascomycota includes many fungi that decompose organic matter and contribute to the nutrient cycle in the rumen ecosystem [3]. Mucoromycota also plays a role in organic material decomposition and is important in the structure and function of the ruminal microbiome [3]. Additionally, the protist analysis indicated that the predominant phyla were Ciliophora and Incertae Sedis, previously documented in primates [60] and humans [47,61]. Ciliophora, characterized by the presence of cilia, are important in predating bacteria and maintaining microbial balance [62].

The increase among butyrate-producing bacteria, notably UCG-010 and UCG-005, across different breeds underscores their crucial role in intestinal health and their potential impact on production traits. These bacteria enhance butyrate production, a key metabolite that supports intestinal barrier integrity, reduces inflammation, and modulates lipid metabolism, processes that are directly linked to intramuscular fat deposition and marbling, critical parameters of meat quality [63,64]. Similarly, the presence of Rikenellaceae RC9 highlights its importance in breaking down fibrous plant materials and facilitating the degradation of complex polysaccharides, including starch, cellulose, and lignin, metabolized in the hindgut, which likely contributes to improved feed efficiency and energy availability for growth and muscle development [65,66,67]. Furthermore, the genus Alistipes, abundantly present across all breeds, plays a multifaceted role in the intestinal microbiome, being associated with protective effects such as enhanced lipid metabolism and reduced inflammation, which are essential for efficient fat deposition. However, it also presents potential pathogenic implications, as it has been linked to colorectal cancer and mental health disorders, underscoring the need for further research to elucidate its precise role in bovine gut health and production [68]. The widespread presence of these taxa across breeds highlights their fundamental contributions to intestinal functionality, influenced by both genetic and environmental factors, and underscores their relevance as potential targets for genetic and dietary strategies aimed at improving health and meat quality in cattle.

Detection of *Clavispora* and *Blastocystis* across all breeds with healthy subjects suggests their potential role as stable components of the gut microbiota, likely influenced by environmental factors [69]. Known for stimulating mucus production through IL-22 and promoting bacterial diversity, *Blastocystis* may have beneficial impacts on intestinal health and immune function [70]. Pichia emerged as one of the most abundant genera in healthy cattle breeds, suggesting its beneficial role in their gut microbiota. Although Pichia has been associated with higher body mass index (BMI) in humans [69], its presence in healthy cattle and alpacas points to a species-specific role in supporting intestinal health without adverse effects [71]. Additionally, Trichostomatia was identified as a prevalent genus in cattle, pigs [49], and other bovines [48], underscoring its significant role in the intestinal microbiota. This aligns with findings of *Trichostomatia* as a diverse and notable component of the gut microbiota in patients with healthcare-associated diarrhea [72]. These consistent observations across different species and health conditions suggest that Trichostomatia plays a crucial role in maintaining a varied and resilient intestinal ecosystem.

Coprococcus exhibited a negative association with meat quality parameters, including GGL and GGC, similar to findings in this study. This suggests that its abundance may negatively influence traits related to fat deposition and marbling [50]. Conversely, Christensenellaceae R-7 demonstrated significant positive correlations with NGM1, NGM2, and GGL, reinforcing its role in lipid metabolism and adiposity traits in cattle. These consistent correlations across studies [73,74] highlight the potential of these genera to influence fat deposition and overall meat quality, underscoring their relevance as targets for microbial modulation strategies. Additionally, Candida demonstrated positive correlations with NGM1, NGM2, GGL, and GGC in cows. While Candida is often associated with infections [75], certain species can act as opportunists without causing disease, as observed in the healthy cows of this study. Notably, Candida may enhance nutrient availability within the gut microbiome, potentially improving fat deposition and meat quality [76]. Furthermore, Candida was identified as a biomarker in Angus cows, aligning with previous reports that Candida glabrata can modulate the ruminal microbiota, initially disrupting microbial balance but ultimately improving milk production in ruminants over time [75]. The predominance of Candida in female cows may reflect a gender-specific microbial profile, as certain species have been reported to exhibit higher prevalence in females [77]. These findings underscore the multifaceted roles of these microbial taxa in cattle, ranging from nutrient metabolism to potential impacts on meat quality. The observed associations highlight the importance of further research to elucidate the mechanisms underlying these relationships and to explore the potential for microbiota-based interventions to optimize animal production and product quality.

The significant negative correlation between Entodinium abundance and meat quality parameters, such as back fat thickness (GGL), hip fat thickness (GGC), and marbling (NGM1 and NGM2), suggests that higher levels of this anaerobic ciliate may detrimentally affect beef quality. This relationship may stem from nutrient competition in the rumen, where Entodinium caudatum primarily metabolizes carbohydrates and feeds on bacteria, producing volatile fatty acids (VFAs) essential for energy production in cattle [78]. While VFAs are critical for ruminal energy metabolism, the degradation of bacterial populations by Entodinium may reduce the availability of microbial protein and other nutrients necessary for intramuscular fat deposition, a key factor influencing marbling and overall meat quality. Additionally, Entodinium caudatum relies on prokaryotic symbionts for survival, highlighting the intricate interplay within the ruminal microbiota. These symbiotic relationships influence ammonia production and interactions with amino acid-fermenting bacteria (AAFB), which are essential for nutrient recycling and conversion efficiency in the rumen. However, an overabundance of Entodinium may disrupt these processes, potentially hindering the deposition of lipids and proteins critical for meat quality traits. Understanding these dynamics provides valuable insights into how microbial competition and metabolic interactions can influence production outcomes, suggesting that targeted modulation of Entodinium populations could be explored as a strategy to optimize meat quality [79].

In Angus cattle, Christensenellaceae R-7 has been identified as a biomarker associated with lean body mass and metabolic health, suggesting its influence on fat deposition and overall meat quality [80]. The presence of Coprococcus as a biomarker in a crossbreed of F1SMxBR cattle suggests that its abundance may be more related to the specific breed characteristics rather than dietary factors, given that all the cattle received the same diet [81]. This aligns with findings indicating that Coprococcus does not appear to be significantly affected by the forage-to-concentrate ratio but may instead be influenced by genetic or environmental variations [73,82]. Such insights highlight the potential role of breed-specific microbial communities in affecting the overall health and productivity of cattle.

The Spearman correlation analysis highlights complex relationships between various alpha diversity indices and hematological and meat quality parameters, underscoring the intricate interplay between the gut microbiota and host physiological traits. These findings are consistent with prior studies that demonstrate the significant role of gut microbiota diversity in influencing both animal health and meat quality [50,83]. The observed negative correlation between GGL and gut microbiota alpha diversity suggests that higher microbial diversity may be associated with reduced intramuscular fat accumulation. This relationship could stem from the enhanced nutrient digestion and fermentation capabilities of a diverse microbiota, which optimize resource allocation for muscle growth rather than fat deposition [84]. Additionally, microbial diversity plays a pivotal role in lipid metabolism by promoting the generation of advantageous metabolites, including short-chain fatty acids (SCFAs) like butyrate, which contribute to intestinal health and systemic metabolic regulation [41]. By improving gut barrier integrity, modulating inflammation, and supporting efficient nutrient absorption, butyrate-producing bacteria may indirectly influence traits associated with meat quality, including fat distribution and marbling. These results emphasize the critical importance of gut microbiota diversity in livestock production. Targeting microbial diversity through genetic selection or dietary interventions could serve as a promising strategy to enhance both production efficiency and meat quality, paving the way for more sustainable livestock management practices.

The alpha diversity indices and negative correlations with hematological parameters, growth, and loin parameters (HGB, WBC, Weight, GL, AL, GPC) underscore the complex role of protists in intestinal health and animal growth, highlighting their potential impact on gut health. Understanding how variations in hematological and meat parameters influence the diversity and functionality of intestinal protists is crucial [85,86].

The correlation analysis indicated that several meat quality parameters are significantly correlated with the beta diversity of bacteria, fungi and protists. These correlations align with recent studies demonstrating that gut microbiota is influenced by host genetic factors [87]. For example, the composition of the rumen microbiota and the function of certain microorganisms are determined by host genetics and can influence feed efficiency and meat quality [73]. Studies have identified genomic regions associated with the abundance of certain rumen bacteria, suggesting that genetic selection could be used to improve ruminal function and feed efficiency in cattle [88].

Ultrasonography is a practical, non-invasive method for evaluating carcass characteristics in live animals; nonetheless, it has certain limitations in accurately capturing all aspects of meat quality compared to direct post-mortem evaluations. Research indicates that ultrasonography can reliably estimate specific carcass attributes, such as muscle area, subcutaneous fat thickness, and degree of marbling [89,90,91]. This makes it a valuable tool in studies where slaughter is not feasible, allowing relevant data on body composition and meat quality to be obtained without compromising animal welfare or genetic resources.

The sample size is a common challenge in microbiome studies as there is no exact formula to calculate statistical power. Various methods, including taxonomic profiling, alpha and beta diversity, and differential analyses, have been proposed to address this issue [92] Certain metrics, such as Shannon’s diversity index and Bray–Curtis dissimilarity, are particularly sensitive to small sample sizes [92,93]. In this study, these metrics revealed statistically significant differences in bacterial, fungal, and protist communities.

## 5. Conclusions

This study underscores the significant influence of breed and controlled dietary management on the gut microbiota composition in cattle, identifying key correlations between microbial genera and critical meat quality traits. Specifically, *Christensenellaceae R-7* and *Alistipes* were positively associated with marbling and muscle area, while *Rikenellaceae RC9* demonstrated a negative correlation with fat thickness. Among fungal genera, *Candida* showed a positive correlation with marbling, whereas *Trichosporon* was negatively associated with muscle depth. The protist *Entodinium* displayed a negative correlation with both fat thickness and marbling. These findings suggest that both genetic and environmental factors shape the gut microbiota in ways that impact meat quality, positioning specific microbial markers as potential indicators for optimizing cattle productivity. While ultrasonography provided valuable in vivo assessments of carcass traits, it has inherent limitations compared to direct post-mortem analyses. This study contributes to advancing microbiota-informed strategies to enhance sustainable cattle production practices, with implications for improving meat quality and animal health management.

## Figures and Tables

**Table 1:** Diet composition and nutritional content of the feeds offered to cattle.

Feed Type	Amount	Dry Matter (%)	Moisture (%)	Crude Protein (%)	Metabolizable Energy (Mcal/kg DM)	Crude Fiber (%)	Calcium (%)	Phosphorus (%)
Corn Silage	10% of body weight	24–26	74–76	7.85	2.25	27	0.29	0.17
Balanced Feed	2 kg	90	10	14.21	2.88	4.75	0.31	0.65

**Table 2:** Composition of the Balanced Feed.

Balanced Feed	%
Ground yellow corn	58.14
Soybean meal	14.53
Wheat bran	23.26
Baking soda	1.57
Common salt	0.5
Mineral salts	2
Total	100

**Table 3:** Average meat quality metrics in different breeds.

Race	GPL	GGL	GL	AL	GPC	GGC	NGM1	NGM2
Angus	6.31	4.12	6.35	55.14	4.00	2.83	162.20	131.16
Braunvieh	6.72	3.32	6.58	61.13	4.51	2.23	107.19	91.71
F1(SMxBR)	6.14	3.18	5.34	46.35	3.56	1.24	83.52	71.16

**Table 4:** PERMANOVA of Bray–Curtis and Jaccard methods.

Bray-Curtis						Jaccard				
**Bacteria**										
	Df	SumOfSqs	R^2^	F	*p-*value	Df	SumOfSqs	R2	F	*p-*value
Race	2	0.61835	0.3339	2.0051	0.001 ***	2	0.8116	0.28707	1.6106	0.001 ***
Residual	8	1.23357	0.6661			8	2.0156	0.71293		0.0003 ***
Total	10	1.85192	1			10	2.8272	1		
	**Fungi**									
	Df	SumOfSqs	R^2^	F	*p-*value	Df	SumOfSqs	R2	F	*p-*value
Race	2	0.25335	0.3723	2.3724	0.0053 **	2	0.48255	0.33902	2.0516	0.0044 **
Residual	8	0.42715	0.6277			8	0.94084	0.66098		
Total	10	0.6805	1			10	1.42339	1		
	**Protists**									
	Df	SumOfSqs	R^2^	F	*p-*value	Df	SumOfSqs	R2	F	*p-*value
Race	2	0.5995	0.34142	2.0737	0.019 *	2	0.79217	0.30164	1.7277	0.0156 *
Residual	8	1.11869	0.65858			8	1.83401	0.69836		
Total	10	1.69864	1			10	2.62618	1		

* *p* < 0.05; ** *p* < 0.01; *** *p* < 0.001.

**Table 5:** Correlation of variables with Beta diversity (Bray and Jaccard) using Mantel and Partial Mantel Tests. Only Significant Variables Presented.

Bacteria	Bray				Jaccard			
	Mantel Test		Partial Mantel Test		Mantel Test		Partial Mantel Test	
Variables	r	*p*	r	*p*	r	*p*	r	*p*
GGL	0.409	0.019	0.408	0.017	0.308	0.028	0.31	0.037
NMG1	0.407	0.007	0.347	0.02	0.328	0.026	0.417	0.003
NGM2	0.376	0.019					0.385	0.016
**Fungi**								
Variables	r	*p*	r	*p*	r	*p*	r	*p*
RBC	0.524	0.01	0.467	0.009				
MCV	0.649	0.012	0.612	0.005				
MCHC	0.697	0.008	0.672	0.004			0.343	0.008
NGM1	0.307	0.035					0.341	0.024
NGM2	0.315	0.02					0.281	0.03
GGC					0.373	0.011	0.38	0.007
GGL								
**Protist**								
Variables	r	*p*	r	*p*	r	*p*	r	*p*
NEU	0.297	0.044	0.293	0.031				
SEG	0.297	0.043	0.293	0.045				
NGM1					0.434	0.001	0.422	0.004
NGM2					0.495	0.001	0.487	0.002
PLT					0.388	0.025	0.379	0.036

## Data Availability

The raw data supporting the conclusions of this article will be made available by the authors upon request.

## Supplementary Materials

The following supporting information can be downloaded at: https://www.mdpi.com/article/10.3390/vetsci11120608/s1, Figure S1: Rarefaction curves of species richness show the sequencing depth of data obtained from the gut samples. Figure S2: Alpha diversity metrics of protists were evaluated in cattle in five breeds. Diversity indices exhibited include ACE, Chao1, Observe, Pielou, Shannon and Simpson. Figure S3. The Venn diagram based on ASVs of cattle fecal microbiota from the five breeds. Figure S4. Relative abundances in the gut microbiota at the phylum and genus level in different cattle breeds. Table S1: Nutritive value of Composition of the Balanced Feed Table S2: List of abbreviations. Table S3: Variables of three breeds groups. Table S4: Kruskal–Wallis results for clinical parameters in cattle by breed. Table S5: Post hoc Bonferroni results for variables in cattle by breed.
