# Supplementary material for: Changes in Gut Microbiota in Peruvian Cattle Genetic Nucleus by Breed and Correlations with Beef Quality
Source: Vet Sci. 2024 Nov 29;11(12):608. doi: 10.3390/vetsci11120608 (PMC11680365; doi:10.3390/vetsci11120608)
Supplement: Supplementary file 1 [file vetsci-11-00608-s001.zip › Supplementary Figures_Quilcate et al.pdf]

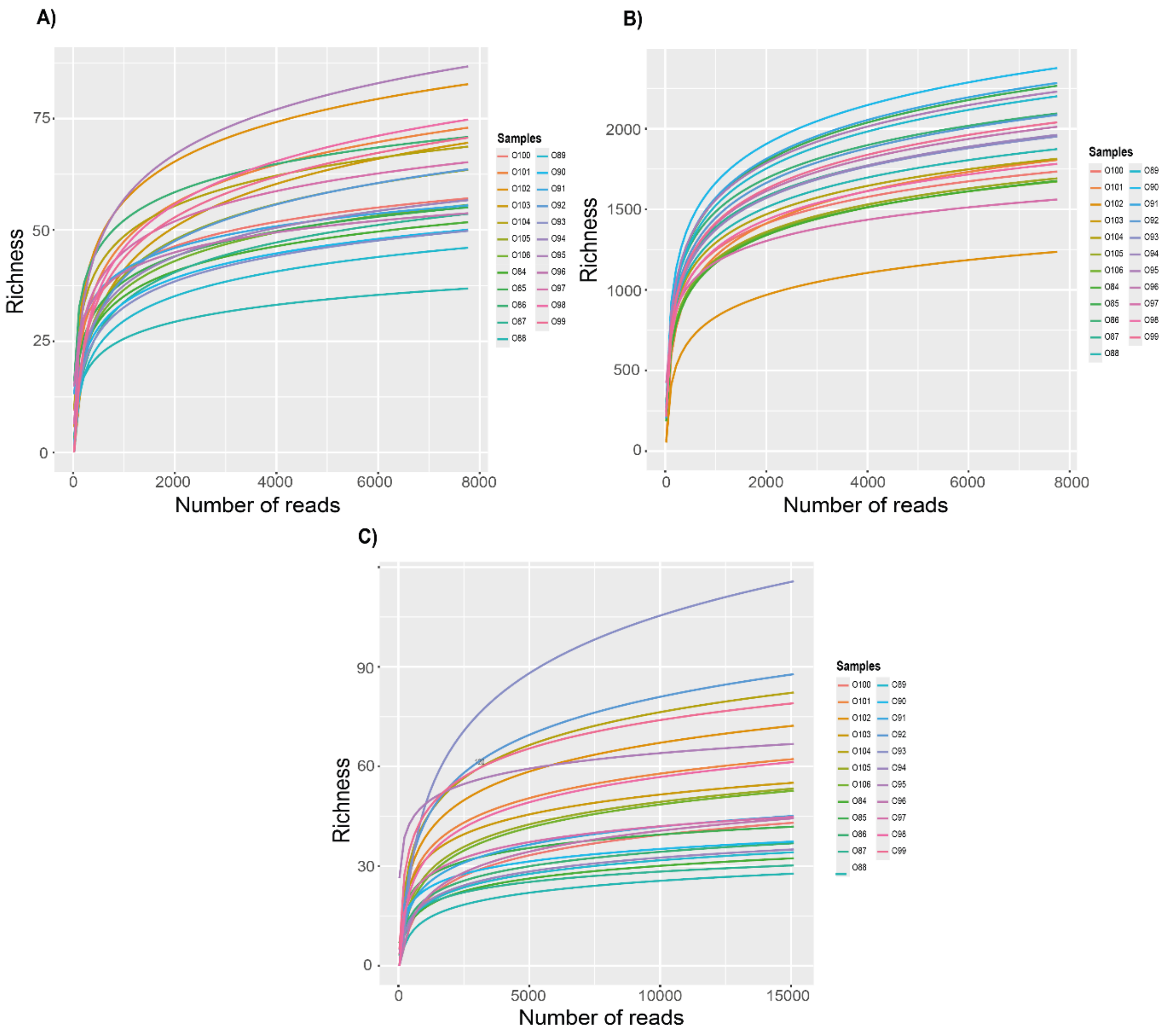

Figure S1. Rarefaction curves of species richness show the sequencing depth of data obtained from the gut samples. A) Rarefaction curves of species richness show the sequencing depth of 16S data obtained from gut samples. B) Rarefaction curves of species richness show the sequencing depth of fungi data obtained from gut samples. C) Rarefaction curves of species richness show the sequencing depth of protists data obtained from gut samples.

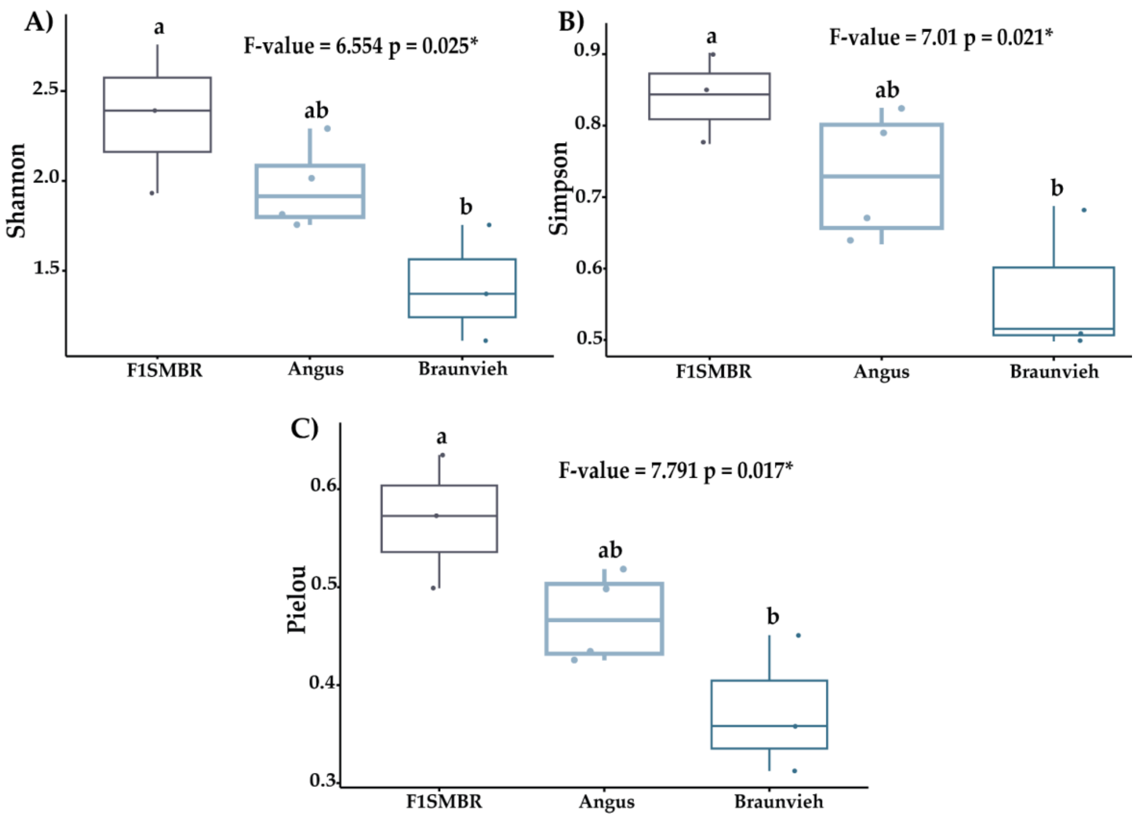

Figure S2. Alpha diversity metrics of protists were evaluated in cattle in three breeds. Diversity indices exhibited include Shannon, Simpson and Pielou.

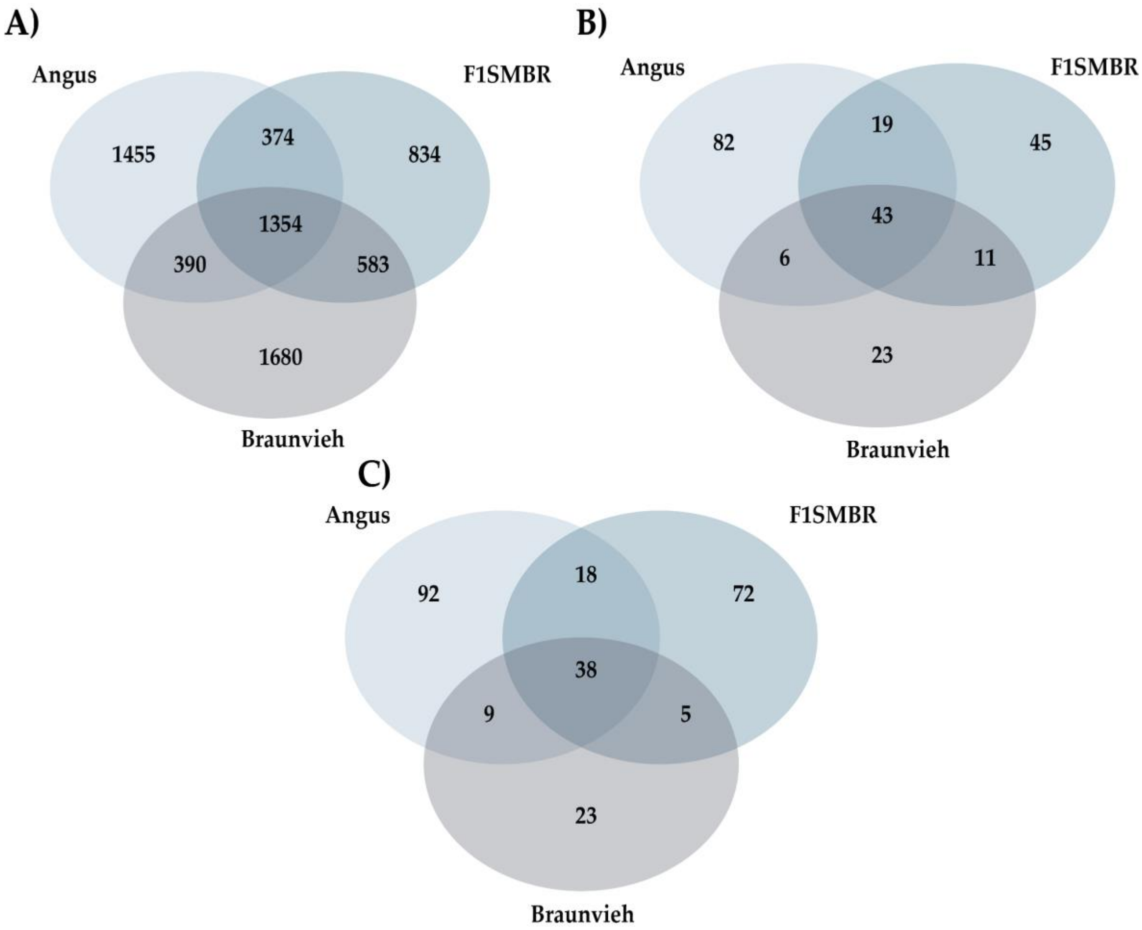

Figure S3. The Venn diagram based on ASVs of cattle fecal microbiota from the five breeds. A) Venn diagram of bacteria. B) Venn diagram of fungi. C) Venn diagram of protists.

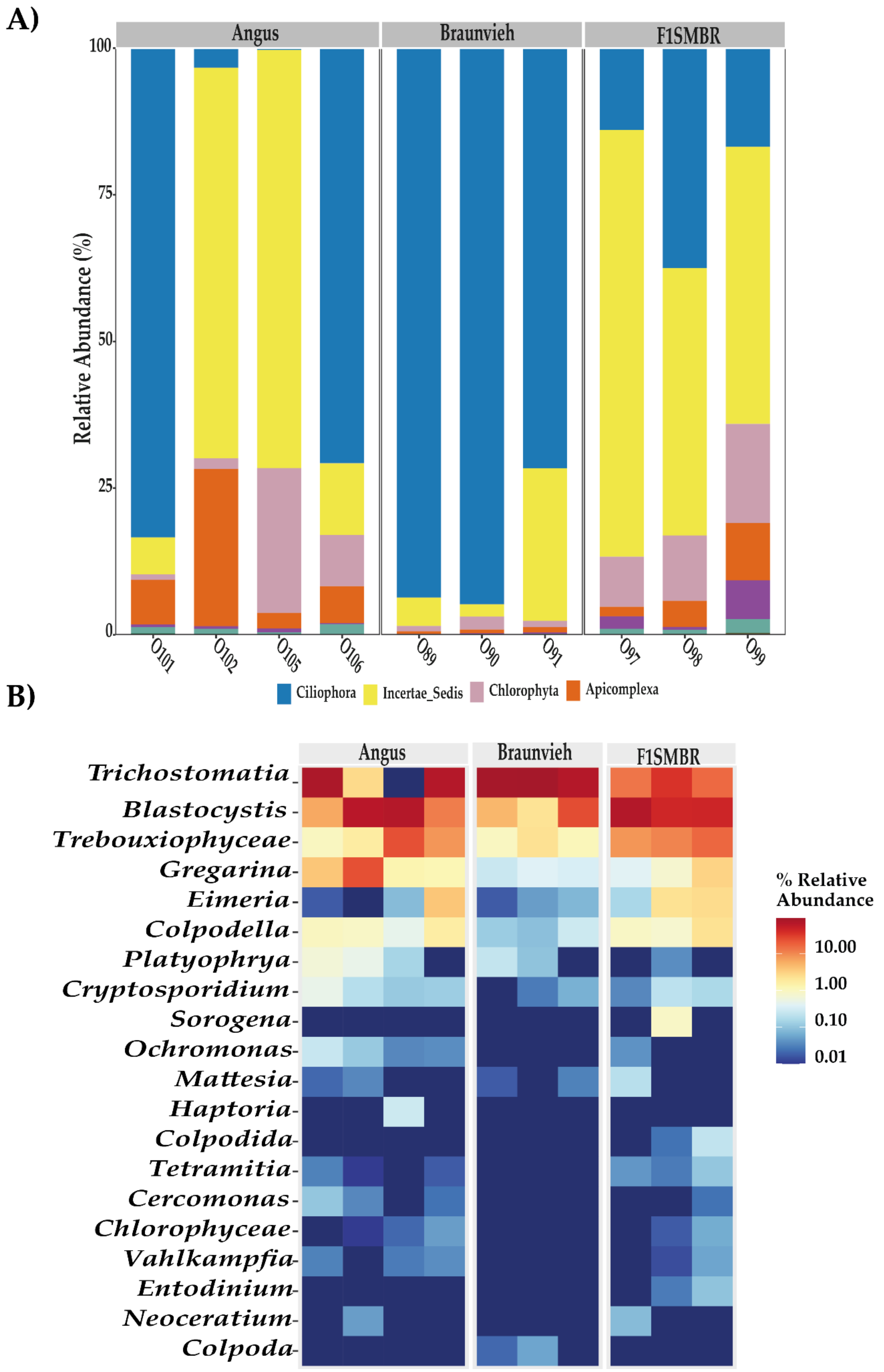

Figure S4. Relative abundances in the gut microbiota at the phylum and genus level in different cattle breeds. A) Bar graph analysis illustrates the abundance of protists phyla in each breed. B) Heat map with the main protists' abundances of the 20 genera in each breed.
